# Improving mechanical properties and electrical conductivity of Al-Cu-Mg matrix composites by GNPs and sc additions

**DOI:** 10.1038/s41598-025-86744-y

**Published:** 2025-01-18

**Authors:** Li Yuan, Fengguo Liu, Chun Wu, Changsheng Lou

**Affiliations:** 1 School of Materials Science and Engineering, Shenyang Ligong University, Shenyang, 110159 China; 2 School of Materials Science and Engineering, Liaoning Technical University, Fuxin, 123000 China; 3 Science and Technology Development Corporation, Shenyang Ligong University, Shenyang, 110003 China

**Keywords:** Al-Cu-Mg-based composites, Scandium (Sc), Graphene nanoplatelets (GNPs), Powder metallurgy, Mechanical properties, Electrical conductivity, Composites, Graphene

## Abstract

To enhance the mechanical properties and electrical conductivity of Al-Cu-Mg-based composites, aluminum matrix composites containing scandium (Sc) and graphene nanoplatelets (GNPs) were fabricated by means of stepwise ball milling, vacuum hot pressing sintering, and hot rolling techniques. When Sc and GNPs were incorporated at concentrations of 0.1 wt% and 0.2 wt% respectively, the resultant composites demonstrated a maximum tensile strength of 326.81 MPa, an elongation of 3.2%, an electrical conductivity of 46.95% IACS, and a hardness of 112.96 HV. In comparison with the 2024 aluminum alloy matrix, enhancements of 39%, 255%, 51% and 51.21% were witnessed in tensile strength, elongation, electrical conductivity, and hardness respectively. These improvements can be primarily ascribed to the addition of Sc, which facilitated the precipitation of solute atoms and enhanced the interfacial bonding between the GNPs and the matrix, as well as the remarkable heterogeneous layered microstructure induced by the incorporation of GNPs. This study presents a feasible approach to concurrently enhance the strength and electrical conductivity of composites through the combined addition of Sc and GNPs.

The development of aluminum-based composites has garnered increasing attention, particularly in the realm of graphene reinforcement. Graphene nanoplatelets (GNPs) are widely utilized in graphene-reinforced aluminum matrix composites (GRMMCs) due to their exceptional electrical conductivity and mechanical properties^1–3^. Composites reinforced with GNPs exhibit not only remarkably high strength^4^ and superior conductivity but also excellent wear resistance^5,6^. Nevertheless, the function of GNPs alone is restricted, and the synthesis of functional GNPs can be intricate and demanding.

Generally, rare earth elements influence the electrical conductivity of aluminum alloys through three primary mechanisms^7–9^. First, they induce lattice distortion in the alloy matrix, leading to scattering of charge carriers and a consequent reduction in conductivity. Second, they refine the microstructure by increasing the density of grain boundaries, where numerous vacancies and dislocations exist, further diminishing conductivity, and third, they can form rare earth active films at the surface of iron-containing phases or react with Al and Fe atoms to form rare earth compounds, thereby effectively reducing the solubility of Fe atoms in the aluminum matrix, which enhances conductivity.

Scandium (Sc), as a rare earth element, significantly improves the high-temperature mechanical properties, microstructural stability, weldability, and corrosion resistance^10,11^ of aluminum alloys with the addition of only 0.1 to 0.4 wt% Sc. Additionally, Sc can purify grain boundaries^12^, resulting in a more uniform microstructure^13,14^. This effect is attributed to Sc’s ability to effectively suppress grain growth in aluminum alloys and promote the precipitation of the θ′ (Al_2_Cu) phase^15,16^. The θ′ phase consists of Al and Cu elements. Under rapid cooling conditions, the uniform distribution of precipitates, such as Al_3_Sc, contributes to the improvement of the alloy’s microstructure^17^. Furthermore, in Al-Cu-Mg alloys, Sc forms Guinier–Preston–Bagaryatsky (GPB) zones, which do not generate significant strain fields, thus reducing electron scattering and enhancing the material’s electrical conductivity. The incorporation of Sc not only strengthens the interfacial adhesion^18–20^ of graphene but also effectively improves the toughness and impact resistance of the composites. Therefore, by adjusting the doping ratio of Sc and graphene, it is expected to synergistically improve the strength and conductivity of GRMMC.

This study investigates the synergistic addition of GNPs and Sc as reinforcing agents. The addition of rare earth elements is typically limited to a maximum of 1.0 wt%, with Sc maintained at 0.1 wt% in this research. Composites with varying GNPs content were prepared using ball milling, hot-press sintering and hot rolling techniques for the 0.1 wt% Sc/Al-Cu-Mg-Sc system. A systematic examination of the microstructure, mechanical properties, and precipitation behavior of the composites was conducted. The approach used to fabricate the composites is schematically shown in Fig. 1.

**Fig. 1:**
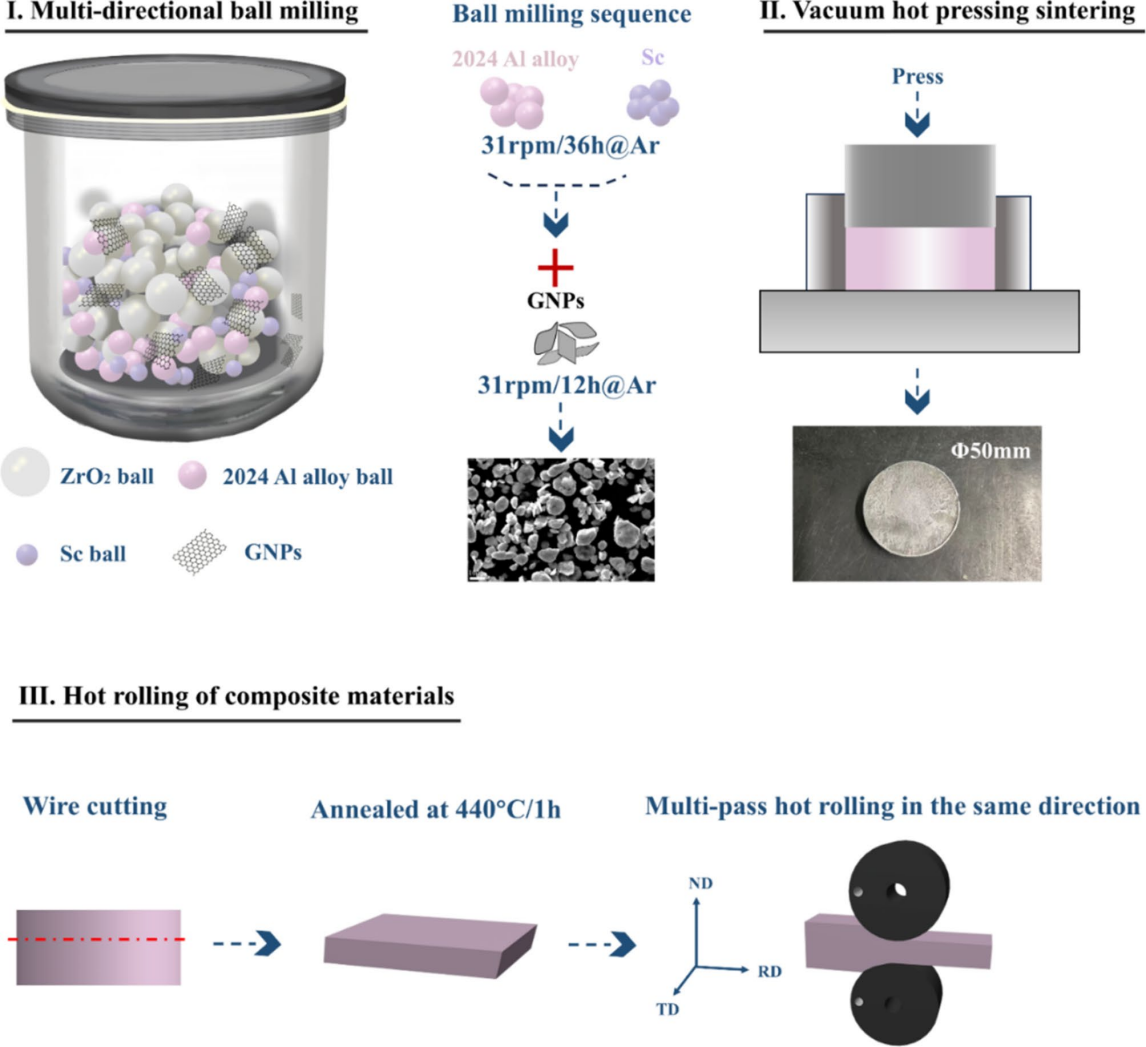
Preparation and processing route of aluminum matrix composites.

The experimental samples are designated as shown in Table 1. The density of the materials was determined using the Archimedes principle, and the relative density was calculated by comparing the measured density with the nominal density of the samples.

**Table 1:** Compositional design and nomenclature of composites.

Sample designation	Al2024 (wt%)	GNPs (wt%)	Sc (wt%)	Nominal density (g/cm^3^)	Relative density/%
0GNPs-U	100(Tabular)	0	0	2.73	99.31 ± 0.07
0GNPs-B	99.9(Tabular)	0	0.1	2.73	99.57 ± 0.13
0.2GNPs-T	99.7(Tabular)	0.2	0.1	2.72	99.24 ± 0.08
0.5GNPs-T	99.4(Tabular)	0.5	0.1	2.72	99.46 ± 0.13
1.0GNPs-T	98.9(Tabular)	1.0	0.1	2.72	99.67 ± 0.21

## Results and discussion

### Microstructure characterization

**Fig. 2:**
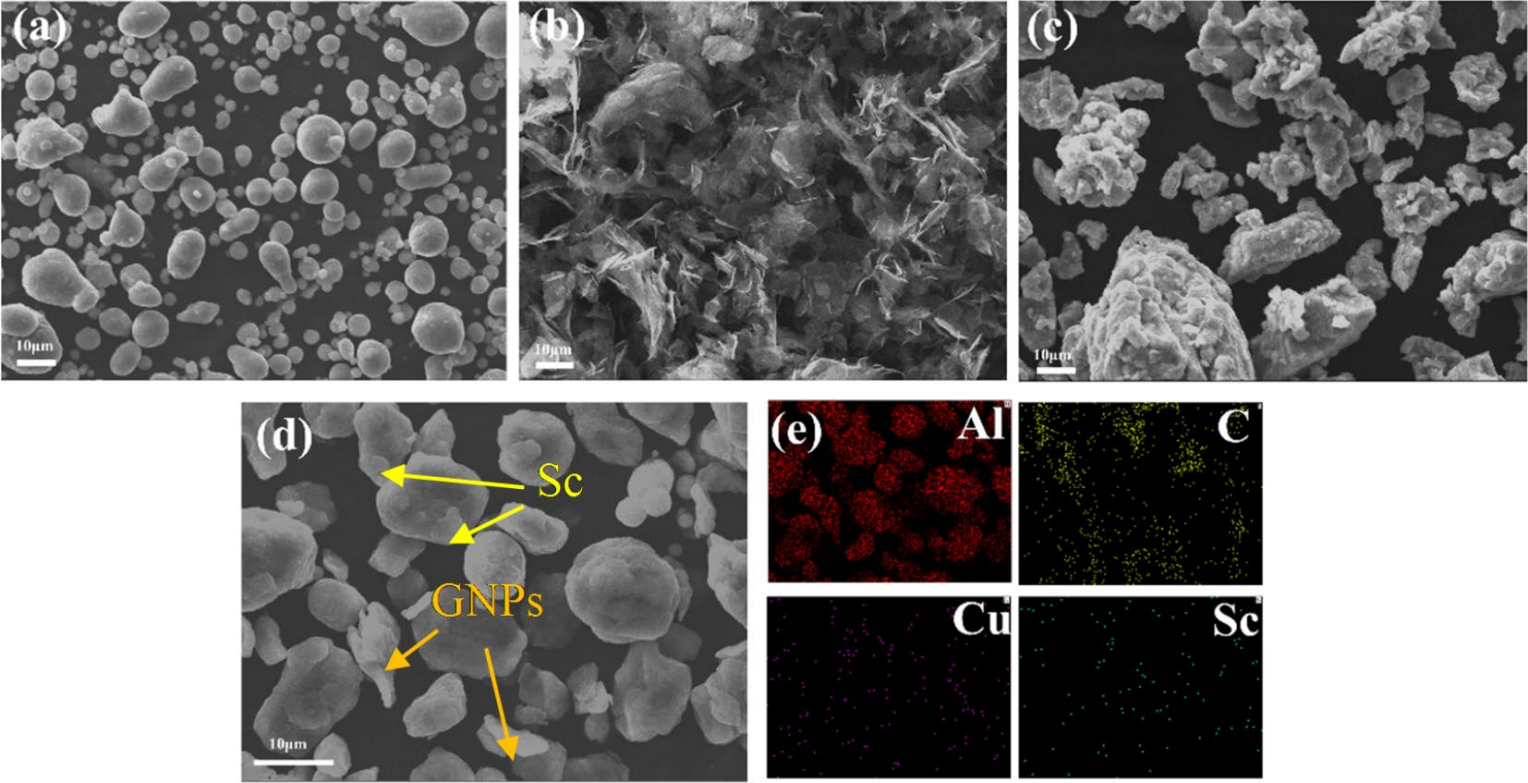
SEM morphologies: (**a**) Al2024 powder, (**b**)GNP, (**c**) Sc powder, (**d**) as-milled powders, (**e**) The corresponding EDS mapping of (**d**).

The SEM images of Al2024 powder, GNPs, and Sc powder are presented in Fig. 2a, b, and c, respectively. The SEM images reveal that the 2024 aluminum alloy powder exhibits a spherical morphology, while the GNPs are characterized by curled, large sheets, and the Sc particles present a polyhedral shape. Figure 2d displays the SEM morphology following ball milling, indicating that the initially spherical Al2024 powder has transformed into irregular polygons, with smaller particles and layered materials interspersed throughout. The corresponding EDS mapping of the Al, C, Cu, and Sc elements is illustrated in Fig. 2e. Based on the EDS mapping presented in Fig. 2e, it can be concluded that the smaller particles and layered materials within the powder comprise Sc and GNPs, suggesting a relatively uniform mixing of the composites.

**Fig. 3:**
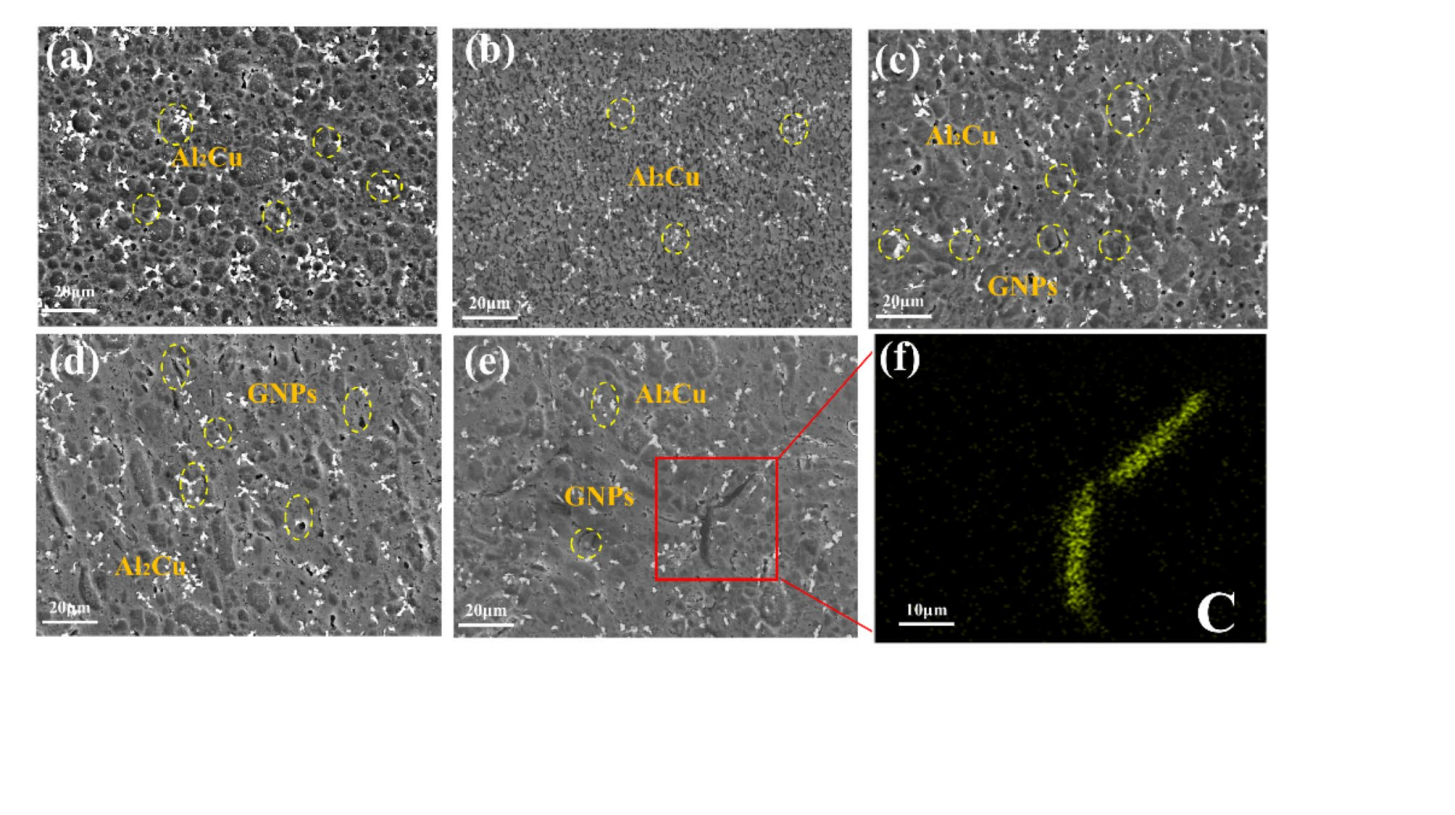
SEM image of the specimen and EDS spectrum of 1.0GNPs-T: (**a**) 0GNPs-U, (**b**) 0GNPs-B, (**c**) 0.2GNPs-T, (**d**) 0.5GNPs-T, (**e**) 1.0GNPs-T. (**f**) EDS of (**e**).

**Fig. 4:**
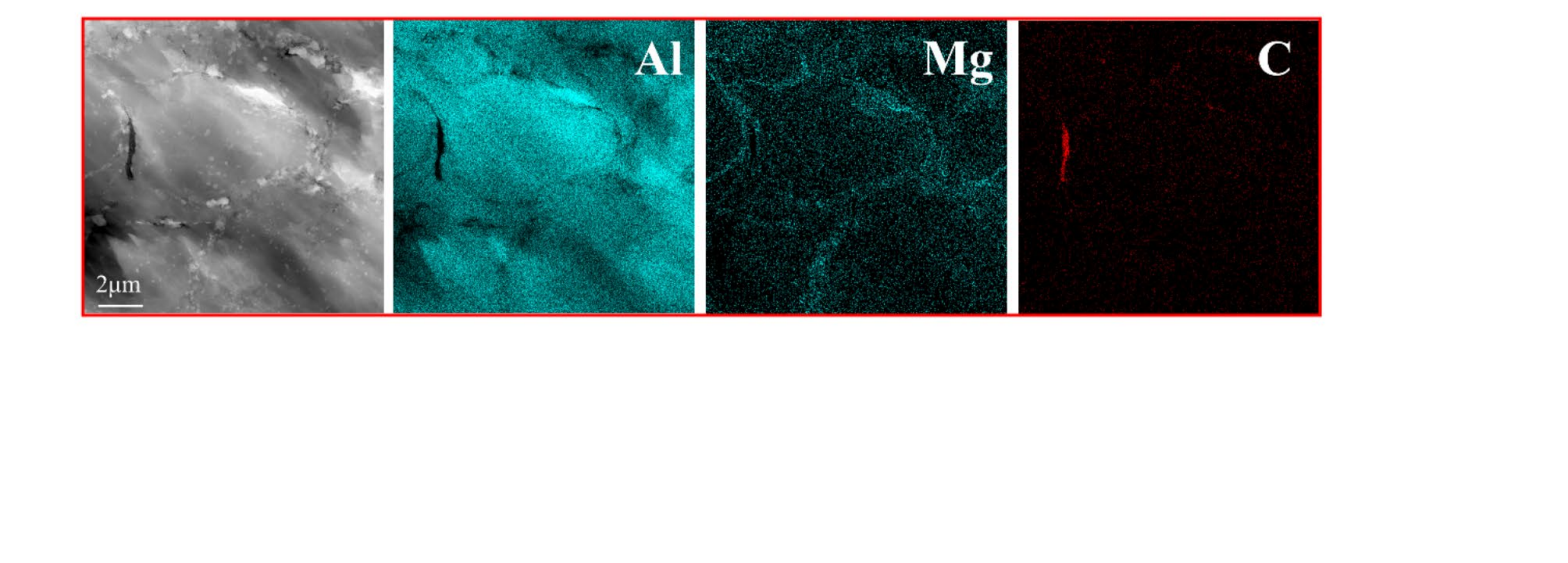
The distribution of GNPs under TEM, along with the EDX spectra for Al, Mg, and C elements.

Figure 3 presents the cross-sectional SEM microstructures of experimental samples with varying mass percentages of GNPs and Sc. The microstructural characteristics of the samples with different reinforcement mass fractions exhibit slight variations, however, significant defects are observed in all cases. As shown in Fig. 3a–e, irregular white phases are consistently identified within the Al2024 matrix. In samples containing GNPs, black elongated structures are also observed. Fig. 3f confirms that these black elongated structures correspond to GNPs. GNPs possess promising electrical conductivity, tensile strength, and self-lubricating properties. The addition of GNPs enhances the electrical conductivity and mechanical performance of the material. However, at higher concentrations, it may lead to a decrease in wear resistance^21,22^. To investigate the distribution of GNPs within the material, we conducted TEM analysis on the samples. The results, shown in Fig. 4, reveal that the GNPs are predominantly located at the grain boundaries^23^.

**Fig. 5:**
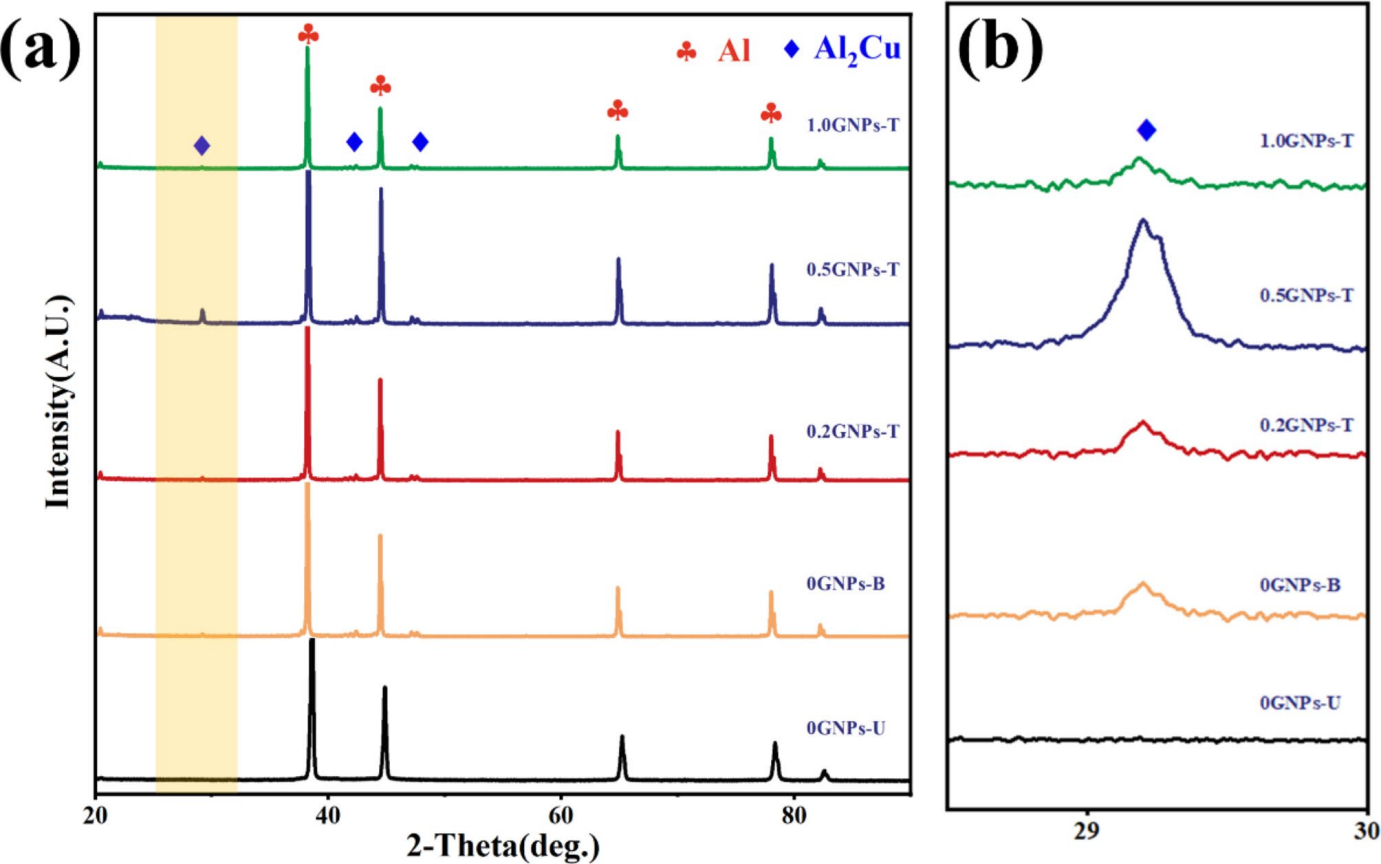
(**a**) XRD patterns of composites with varying Sc/GNPs content, (**b**) Enlarged view of the yellow area in Fig. 4a.

**Fig. 6:**
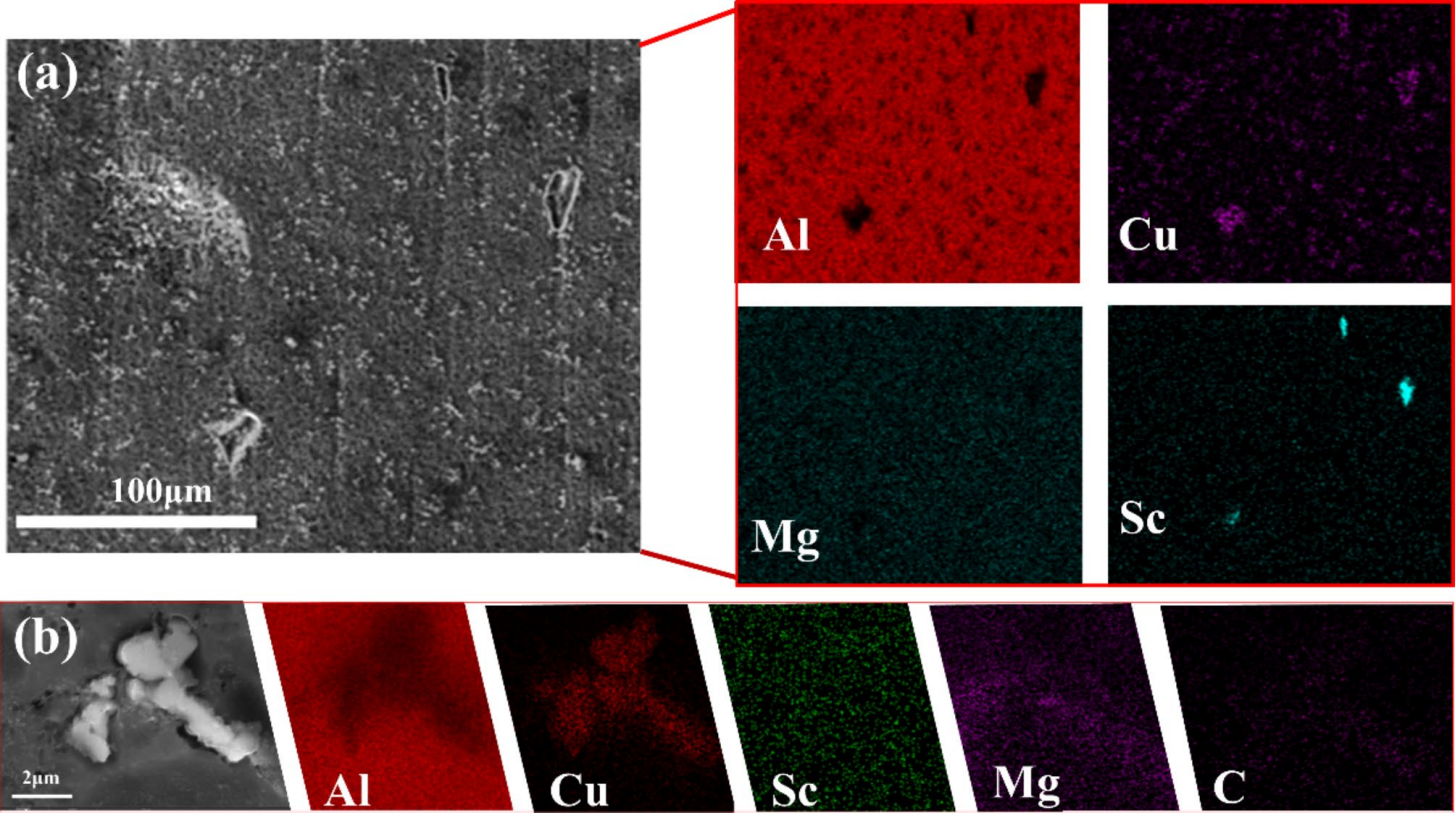
(**a**) SEM image of 0GNPs-B and corresponding EDS plot, (**b**) SEM plot and EDS plot of Al_2_Cu.

The XRD patterns of the samples after hot rolling are presented in Fig. 5. The results indicate that the grain structure undergoes a significant transformation upon the addition of 0.1 wt% Sc. Specifically, distinct diffraction peaks corresponding to the θ’ phase appear in the XRD spectra upon incorporating 0.1 wt% Sc into the matrix material. Figure 6a displays the SEM image and corresponding EDS spectrum for the 0GNPs-B sample, where a notable enrichment of Cu is observed in the regions surrounding Sc. Figure 6b illustrates the SEM image and EDS spectrum of the irregular white phase present in the 0.2 GNPs-T sample. When combined with the XRD analysis, it can be concluded that the white phase identified in Fig. 3 corresponds to the θ′ phase, which is known to significantly enhance the strength of aluminum alloys through precipitation hardening mechanisms. Furthermore, the θ′ phase is detected in all samples, however, the intensity of the diffraction peaks associated with the θ′ phase increases with the addition of Sc. This observation, along with the findings in Fig. 6a, suggests that the inclusion of Sc promotes the precipitation of the θ′ phase within the Al2024 matrix. However, the peaks corresponding to Sc, GNPs, and Al_3_Sc are not observed in the XRD patterns, likely due to their low concentrations.

**Fig. 7:**
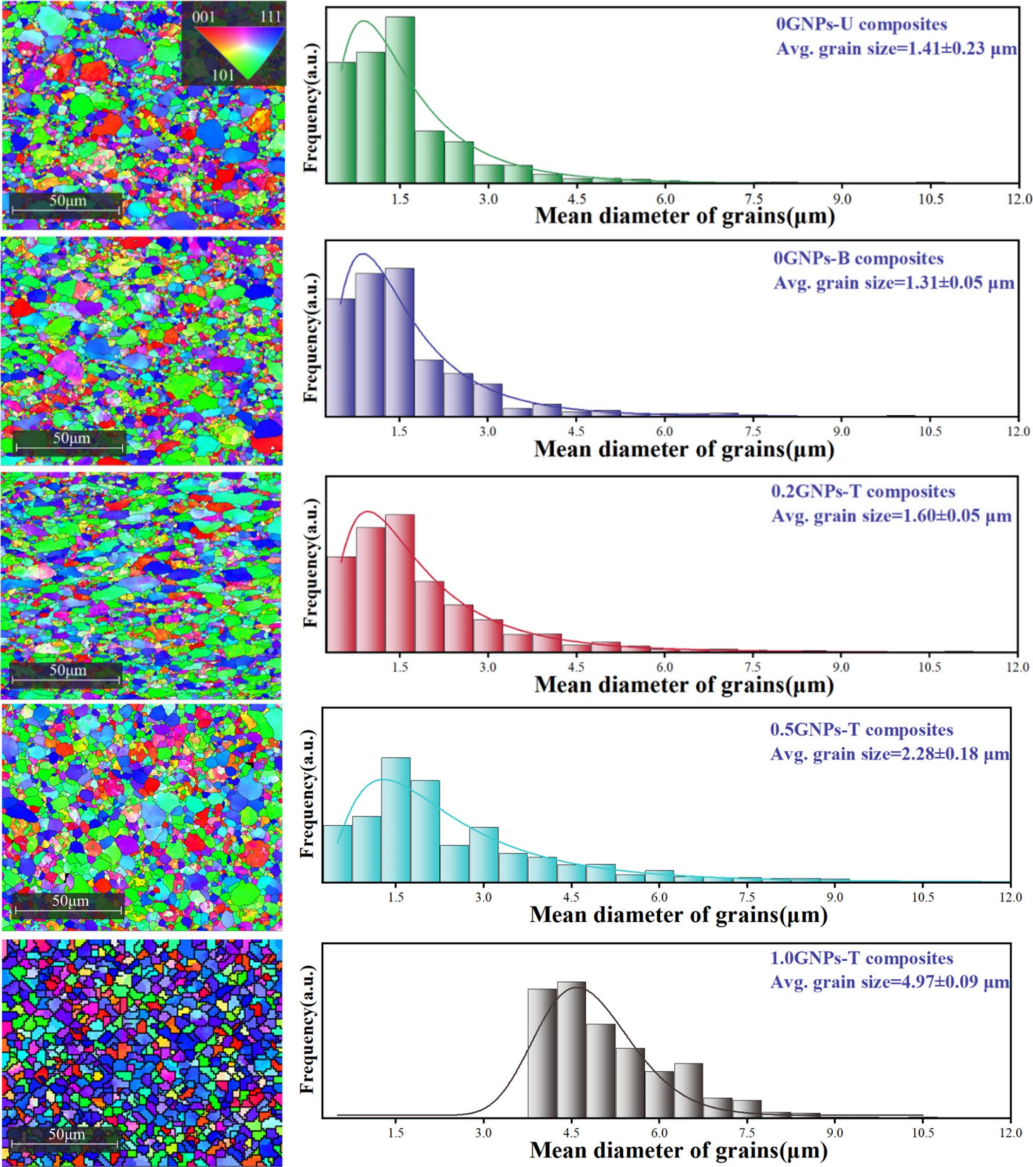
EBSD results including IPF maps and grain diameter distribution of composites.

**Fig. 8:**
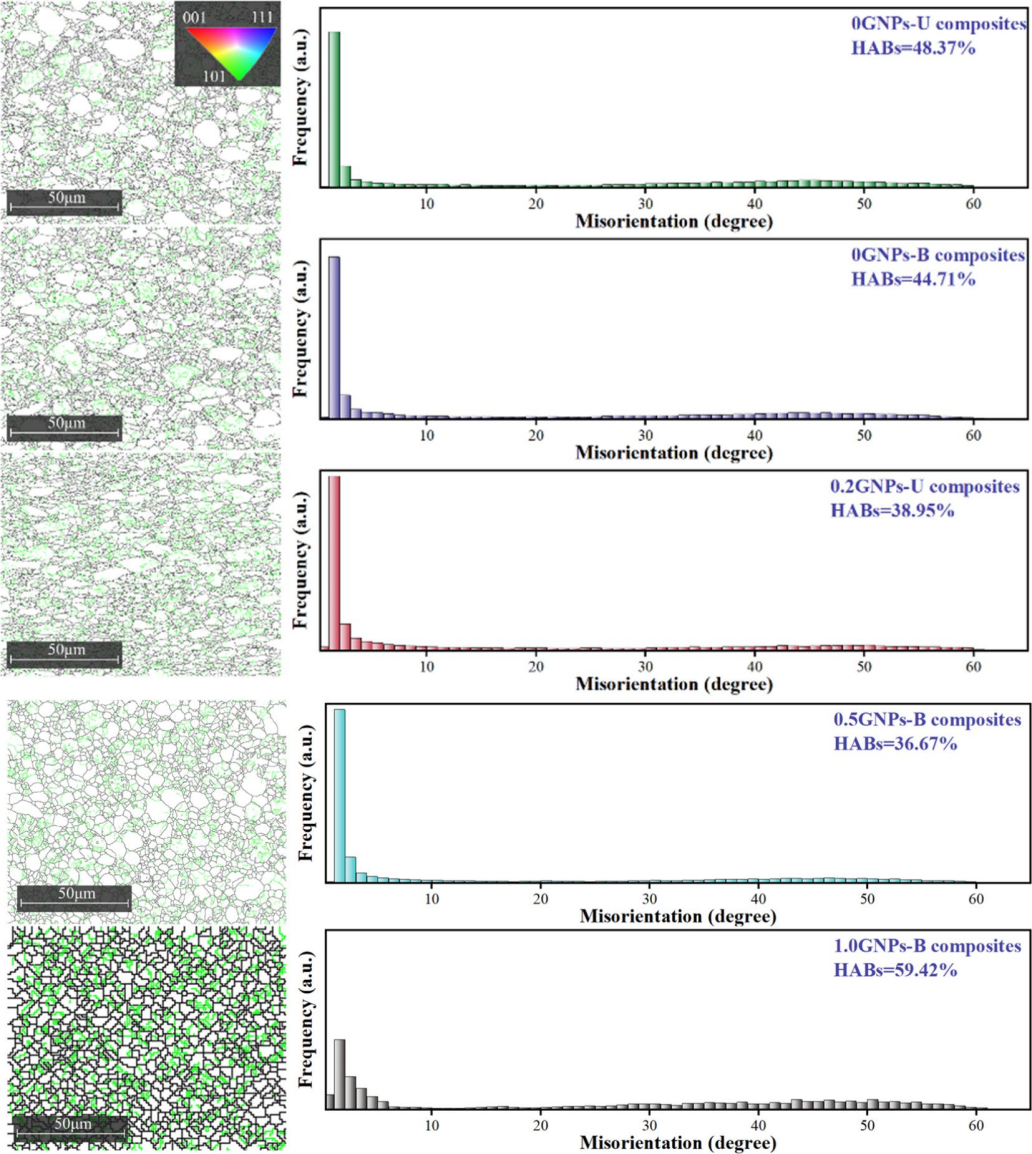
EBSD results of the distribution of large and small angular grain boundaries.

Figure 7 and 8 show the EBSD results of the composites samples. All inverse pole figures (IPF) are obtained from the cross-sections of the samples after hot rolling. The IPF and grain size distribution are presented in Fig. 7. The primary orientation of the 2024 aluminum alloy is {111}, while the main orientation of the composites is {101}, which is consistent with the XRD analysis data. The grain size distribution indicates that the addition of Sc to the composites refines the grain structure compared to the 2024 alloy, suggesting that Sc addition promotes dynamic recrystallization in the composites. Figure 8 presents the distribution of low-angle grain boundaries (LAGBs) and high-angle grain boundaries (HAGBs). Green lines represent LAGBs, while black lines represent HAGBs. LAGBs are concentrated around small-sized grains, whereas HAGBs are mainly located between columnar grains. The results indicate that the addition of GNPs and Sc increases the content of LAGBs. LAGBs, as a form of lattice defect, exhibit higher dislocation density in their aggregation regions, which can hinder dislocation movement and enhance the mechanical properties of the material^24^.

The EBSD analysis further reveals that when GNPs are added, the higher hot-pressing temperature and extended holding time, combined with the excellent thermal conductivity of GNPs, promote atomic diffusion and grain boundary migration, leading to abnormal grain growth. Additionally, the increased dislocation density associated with LAGBs provides extra energy, causing the grains to coarsen during thermal processing^25^.

### Hardness and frictional behavior

**Fig. 9:**
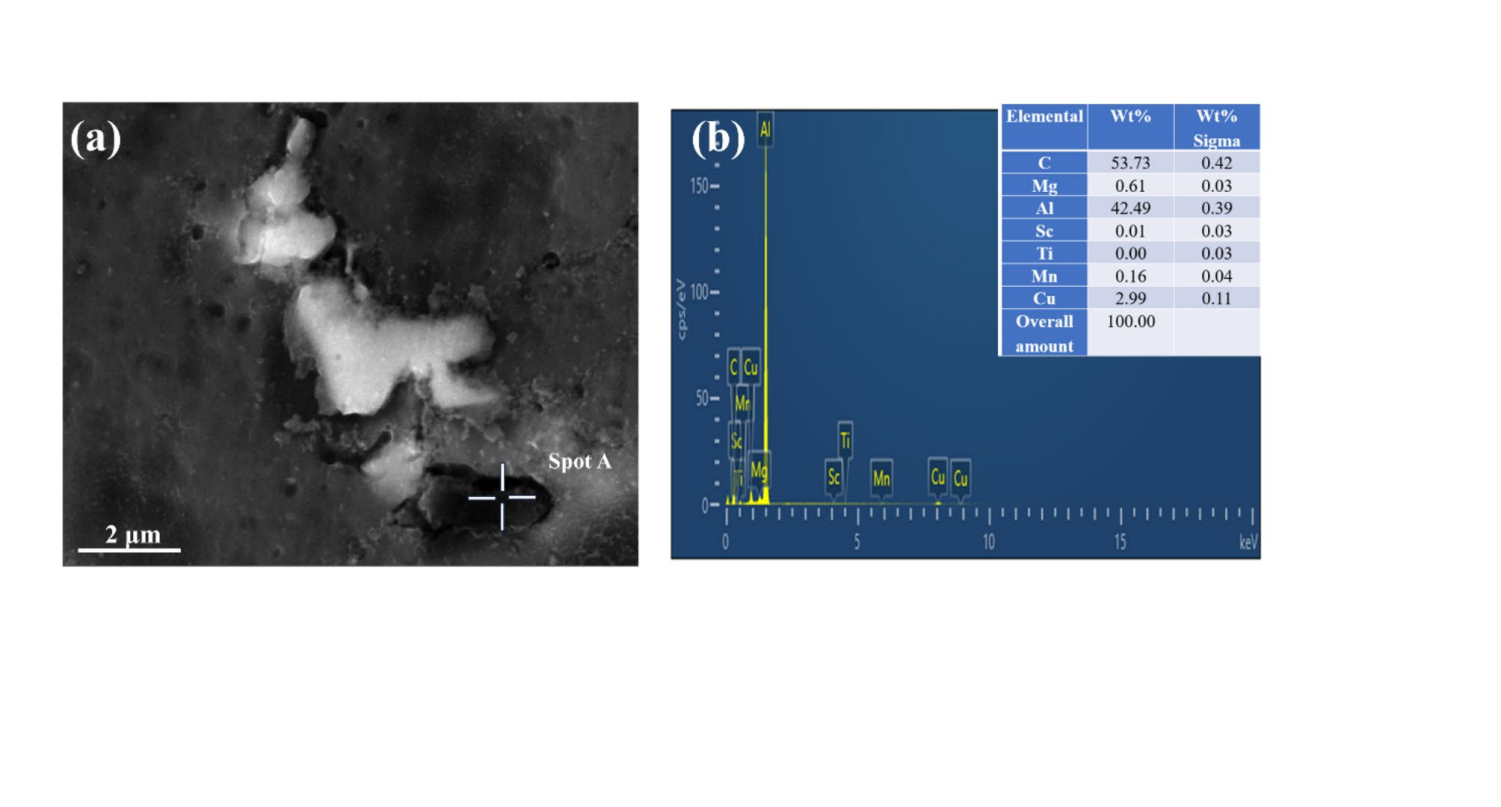
(**a**) SEM plot and EDS dot scan plot of 1.0GNPs-T, (**b**) EDS results of the Al_4_C_3_ in (A).

**Fig. 10:**
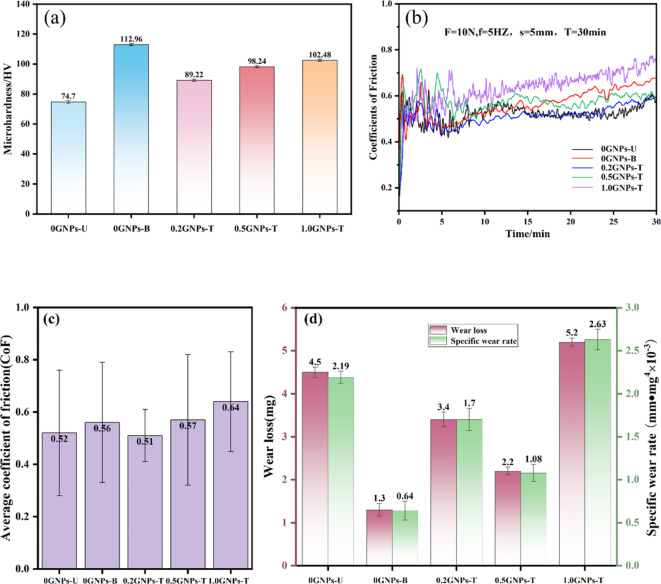
Hardness and wear properties of composites.

Figure 9 presents the SEM image and EDS point scan results for the 1.0GNPs-T sample. The EDS point scan results for region A reveal that the black block phase corresponds to Al_4_C₃, which has been shown to significantly enhance the strength and hardness of materials^26,27^. The hardness of the composites is illustrated in Fig. 10a. Notably, the addition of 0.1 wt% Sc has a pronounced effect on the hardness of the composite, increasing it from 74.7 to 112.96 HV, representing an improvement of 51.21%. This result provides strong evidence of the beneficial impact of Sc on the Al2024 matrix. Although the hardness of the composites decreases with the initial incorporation of GNPs compared to the addition of Sc alone, further increments in GNP content leads to an increase in hardness, achieving a value of 102.48 HV. This represents a 37.18% enhancement over the Al2024 matrix, attributed to the increased formation of Al_4_C_3_ due to the higher GNP concentration. This finding underscores the synergistic effects of Sc and GNPs on the properties of Al2024. Furthermore, in accordance with dislocation strengthening theory^28–30^, the dispersed GNPs are effectively pinned at grain boundaries, which inhibits grain growth and enhances the Hall-Petch strengthening mechanism and microstructural uniformity of the composites^31^, thereby further increasing the hardness.

The coefficient of friction (CoF, µ) of GNPs-Sc/Al2024 composites as a function of sliding time is illustrated in Fig. 10b. The friction curve was obtained under a load of 10 N, with a friction frequency of 5 Hz and a single reciprocating distance of 5 mm. All samples exhibited a pronounced initial increase in CoF, followed by a decrease, indicating that the GNPs did not significantly influence the friction behavior of the materials during the early stages of wear. After approximately five minutes of running-in, the surface developed uneven protrusions, resulting in fluctuations in CoF^32^. However, as wear continued, the temperature of the matrix material increased, enhancing adhesion at the friction interface and leading to a rise in CoF. When the GNPs content was 0.5 wt% and 1.0 wt%, the increased CoF could be attributed to the agglomeration of graphene. This agglomeration contributed to an increase in surface roughness and porosity of the material.

Figure 10c presents the average CoF of the composites, revealing that the addition of GNPs did not reduce the CoF of the material. This phenomenon may be associated with the presence of the θ’ phase, which could induce an uneven stress distribution^33–35^. Such uneven distribution may result in stress concentration during friction, thereby promoting the occurrence of fatigue wear and adversely affecting the friction performance of the material.

Figure 10d illustrates the wear volume and specific wear rate of the composites. This data reflects the actual wear behavior of the composites. Notably, the wear rate was significantly reduced when only 0.1 wt% of Sc was added, attributed to the increased hardness of the material, which enhanced its wear resistance. In the friction process, higher hardness allowed the material surface to better withstand wear, consequently lowering the wear rate. It is important to note that when the GNPs content reached 1 wt%, both the wear rate and average CoF reached their maximum values, which may also be linked to the agglomeration of graphene. The agglomeration of GNPs increased the surface roughness and porosity of the material^36^, thereby diminishing its wear resistance.

**Fig. 11:**
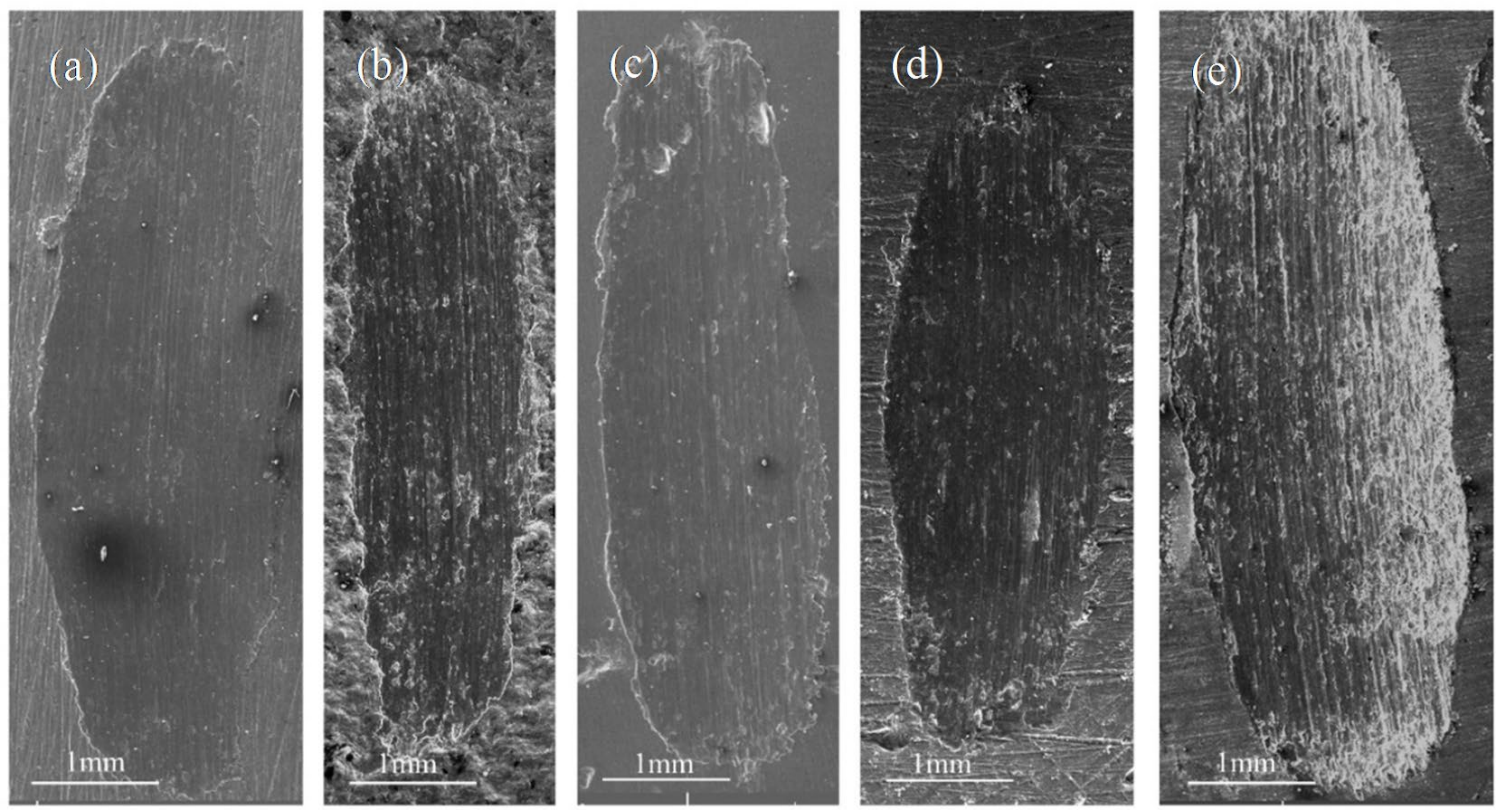
Secondary electron SEM images of the wear track: (**a**) 0GNPs-U. (**b**) 0GNPs-B. (**c**) 0.2GNPs-T. (**d**) 0.5GNPs-T. (**e**) 1.0GNPs-T.

**Fig. 12:**
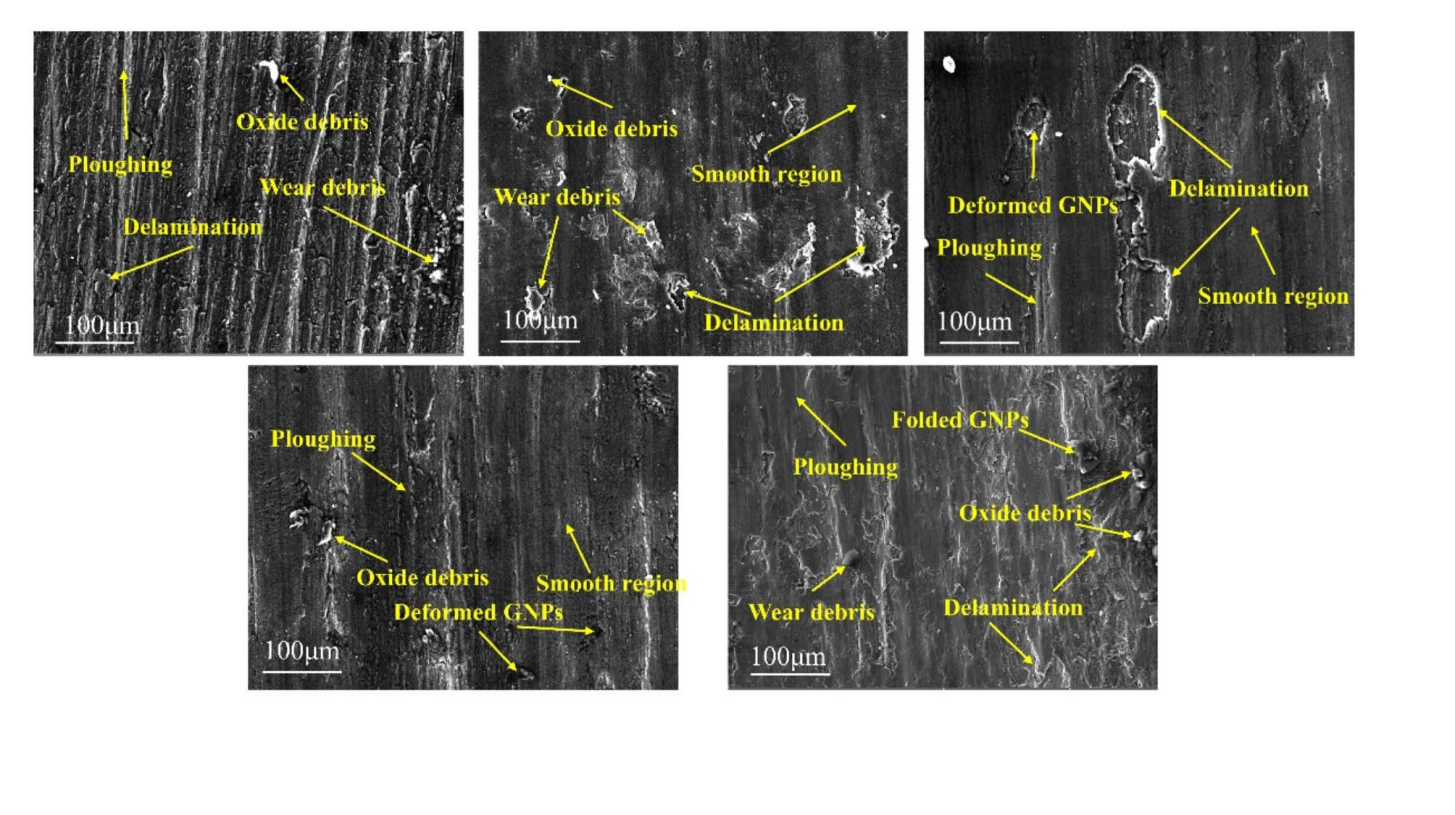
SEM images of worn surfaces of composites: (**a**) 0GNPs-U. (**b**) 0GNPs-B. (**c**) 0.2GNPs-T. (**d**) 0.5GNPs-T. (**e**) 1.0GNPs-T.

The overall wear morphology of the GNPs-Sc/Al2024 composites is shown in the SEM images in Fig. 11. The wear tracks of all tested samples exhibit an uneven wear pattern. The SEM images of the wear surfaces of the GNPs-Sc/Al2024 composites are shown in Fig. 12. The wear surfaces of the samples all display micro-plowing grooves along the sliding direction, indicating that abrasive wear occurred in all the samples during the testing. Oxides and high-hardness carbides, such as Al_4_C_3_^37^, were generated during the sliding process. These substances are able to either slide or roll freely on the contact surface of the sample, resulting in abrasive wear. From the images, it can be seen that the wear surface of the 2024 aluminum alloy displays deep and long plowing grooves, accompanied by a deformation layer formed along the sliding direction. When only Sc is added or when both Sc and GNPs are added, the number and depth of the plowing grooves are significantly reduced. The addition of GNPs leads to an increase in the smooth areas on the wear surface. However, when the GNPs content reaches 1.0 wt%, the wear surface of the composites exhibits increased delamination and material spalling as shown in Fig. 11e.

**Fig. 13:**
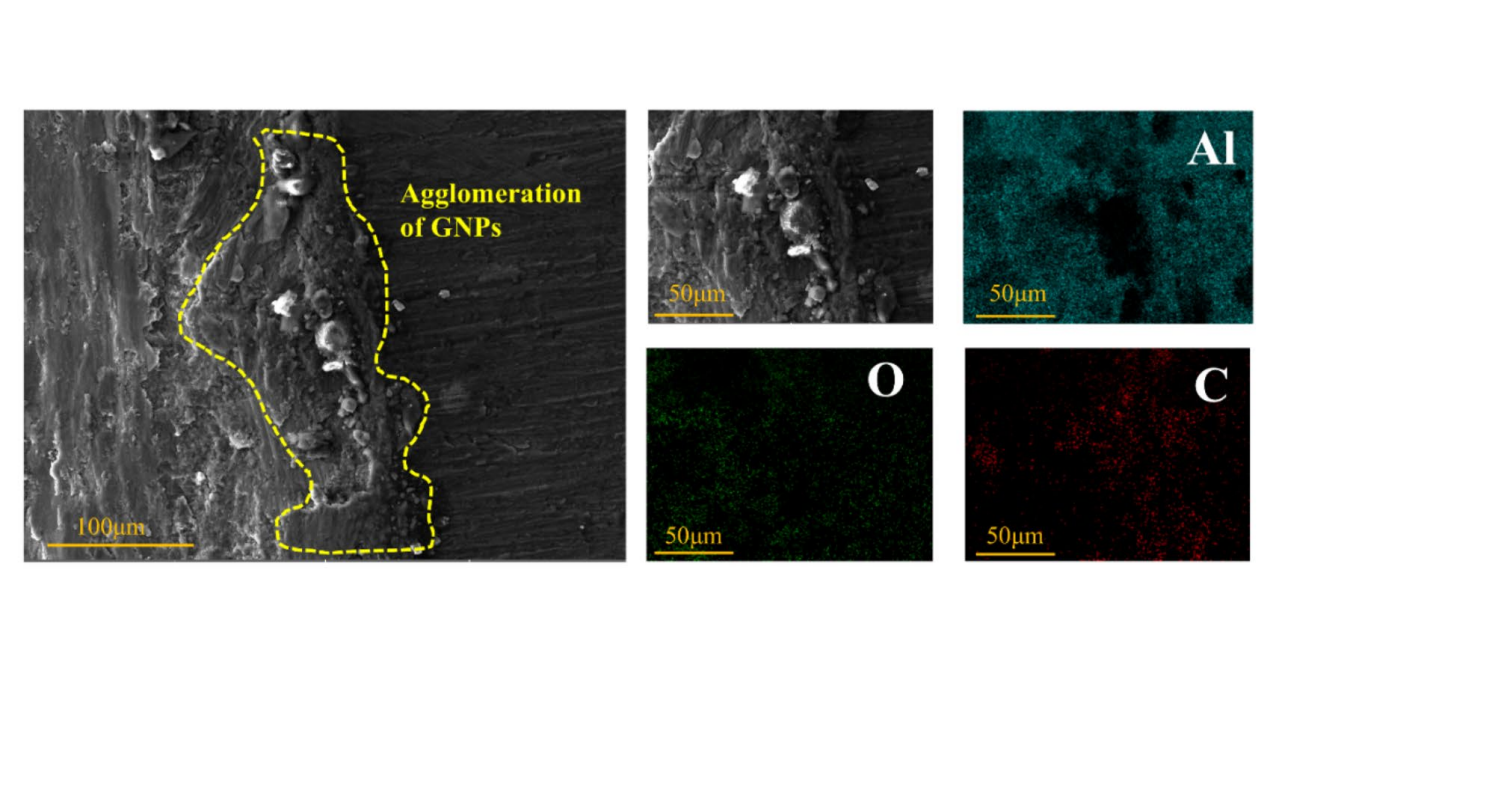
SEM image and corresponding EDS image of the 1.0 GNPs-T wear edge.

**Fig. 14:**
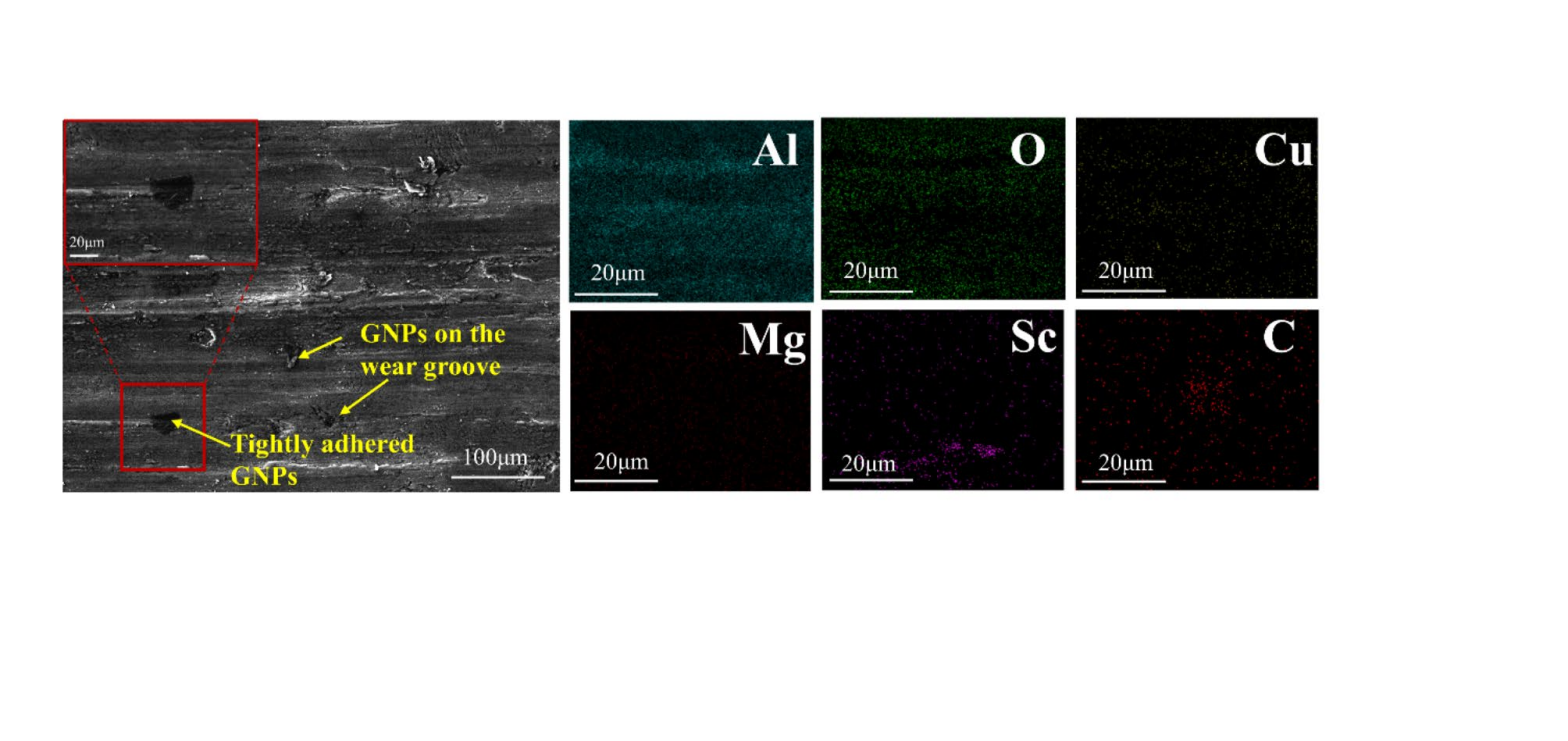
SEM image and corresponding EDS image of the 0.5 GNPs-T wear edge.

Figure 13 shows the accumulation of wear debris at the wear edge and the corresponding EDS mapping for the composite with 1.0 wt% GNPs. It can be observed that, at this GNPs content, the spalled matrix material consists mainly of GNPs and carbides. This phenomenon is primarily attributed to the aggregation of GNPs within the composite, which increases the porosity. The clustering of graphene negatively impacts wear resistance by acting as a stress concentration zone. In contrast, when the GNPs content is low and the distribution is uniform, the GNPs are tightly bonded to the matrix. In the process of friction, the GNPs present in the plowing grooves help to reduce friction and wear on the material surface owing to their inherent self-lubricating properties^38^, as depicted in Fig. 14.

**Fig. 15:**
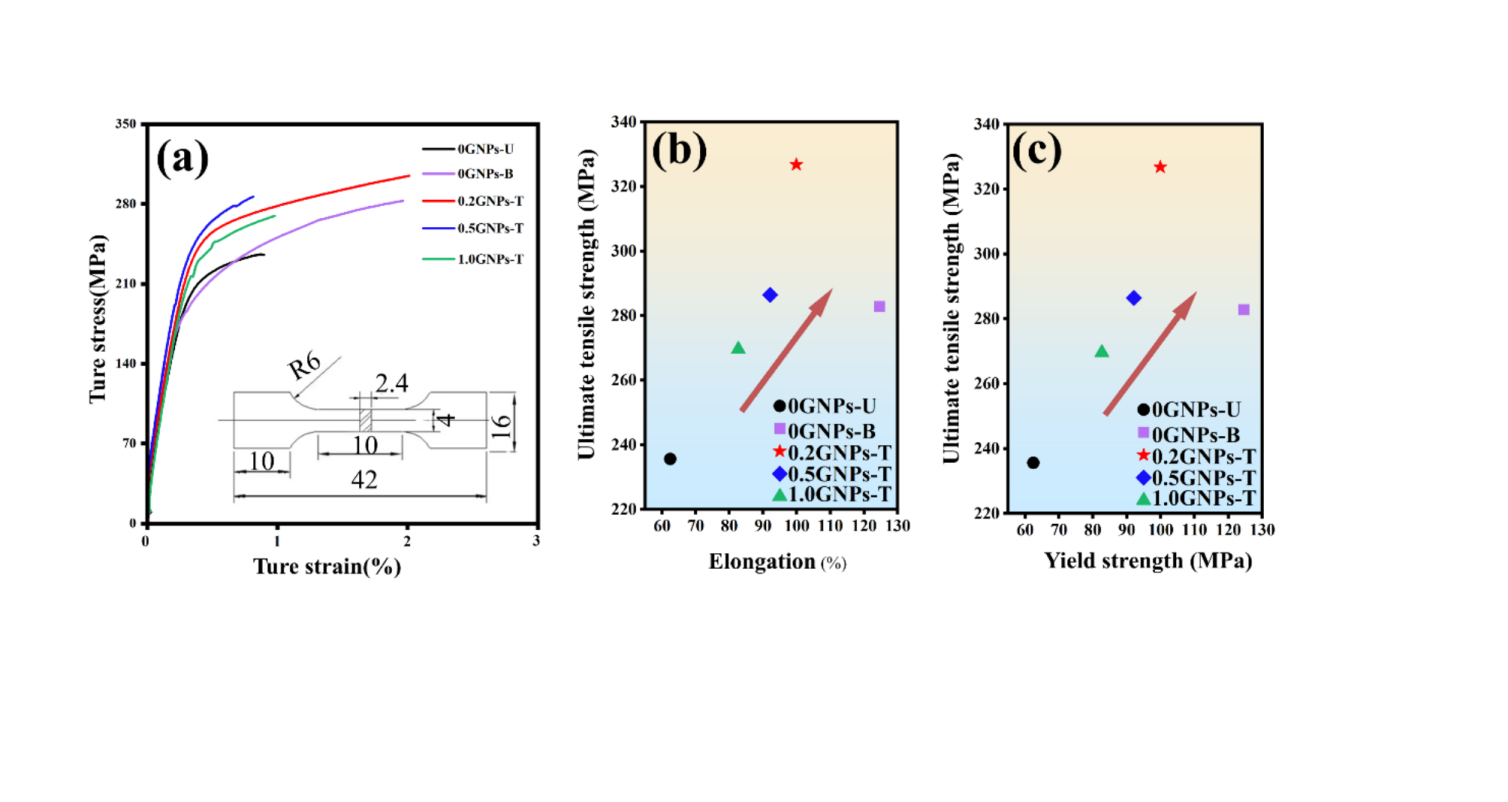
Mechanical and electrical properties of composites after rolling: (**a**) Tensile ture stress-strain curves after rolling, inset exhibits the dog-bone appearance of tensile specimens, (**b**) Comparison of elongation and ultimate tensile strength among different samples, (**c**) Comparison of yield stress and ultimate tensile strength among different samples.

Figure 15 illustrates the mechanical properties of Al2024 composites with varying contents of GNPs and Sc. Key parameters of the mechanical properties are summarized in Table 2. The elongation at break of the composite containing Sc reached 2.0%, which represents an increase of 122%, while the ultimate tensile strength increased by 47.31 MPa, a growth of 20%, compared to the composites without Sc. These results indicate that Sc plays a significant role in toughening and strengthening the composites. The true tensile strength variations of the 2024 aluminum alloy and its composites are presented in Fig. 15a. It is evident that the yield strength of the composites was significantly enhanced through the incorporation of GNPs and Sc, showing an increase of 32–99% over the unmodified 2024 aluminum alloy. Furthermore, it can be observed from the figure that the composite exhibits the maximum ultimate tensile strength of 326.81 MPa at a GNPs content of 0.2%, which is an improvement of 38.76% compared to the 2024 aluminum alloy. Figure 15b, c depict the relationships between ultimate tensile strength and elongation, as well as ultimate tensile strength and yield strength for the tested materials. At a GNPs content of 0.2 wt%, the composite achieves maximum values for both ultimate tensile strength and elongation, exceeding the base material by 38.76% and 255.55%, respectively. These findings clearly demonstrate that the simultaneous addition of GNPs and Sc leads to a marked enhancement in the mechanical properties of the composites.

**Fig. 16:**
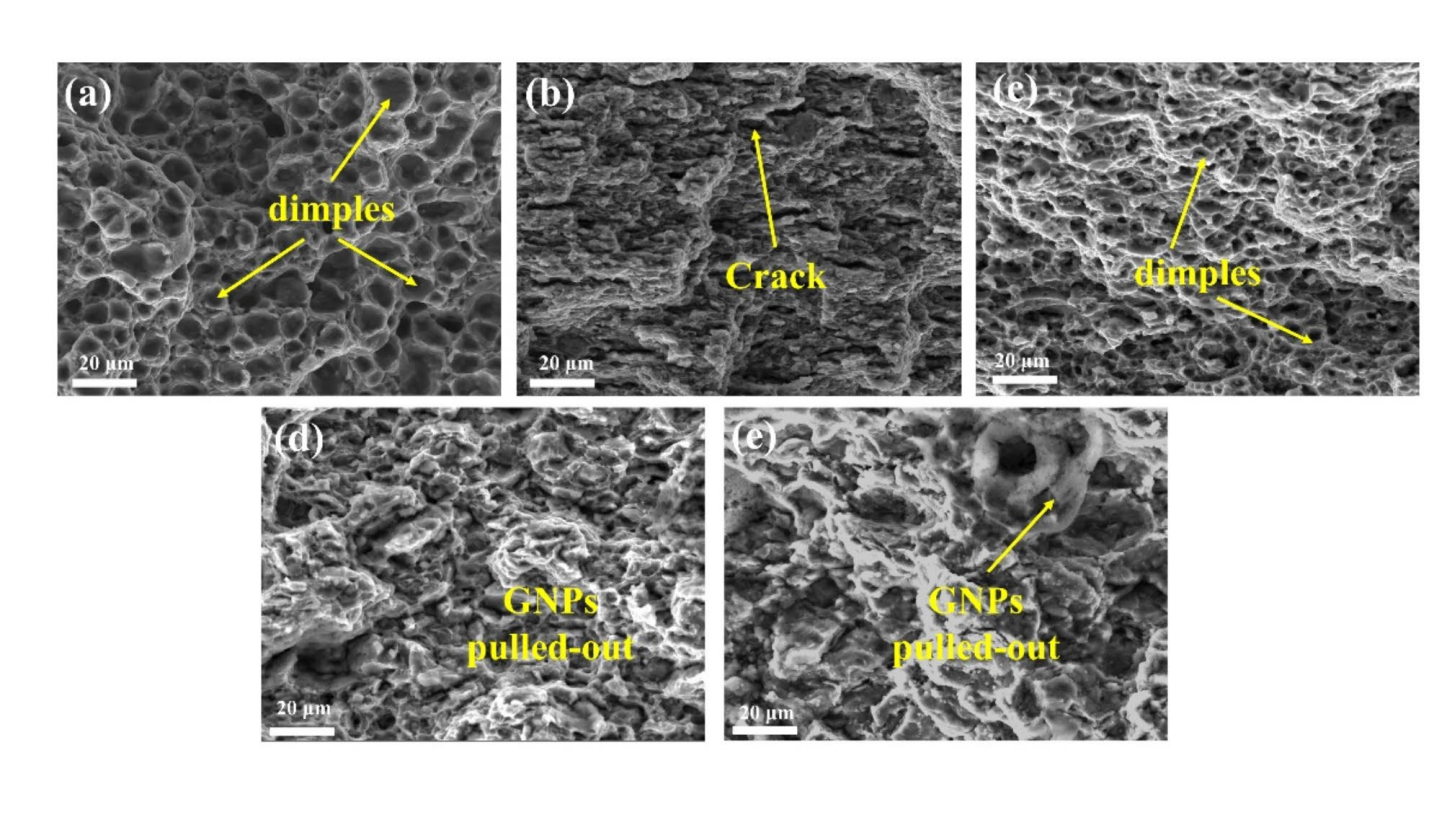
Tensile fracture morphology and fracture schematics of composites: (**a**) 0GNPs-U, (**b**) 0GNPs-B, (**c**) 0.2GNPs-T, (**d**) 0.5GNPs-T, (**e**) 1.0GNPs-T.

Figure 16 illustrates the fracture morphology of GNPs-Sc reinforced Al2024 composites. The results indicate that the fracture mode of Al2024 is characterized by ductile rupture, while the tear types for the 0GNPs-T, 0.5GNPs-T, and 1.0GNPs-T samples exhibit a different form of particle fracture. The fracture mode for the 0.2GNPs sample is a combination of ductile rupture and particle fracture. As shown in Fig. 16a, the fracture surface of the 2024 aluminum alloy displays shallow dimples extending along the loading direction, suggesting low ductility. In the case of the 0GNPs-B composite, Fig. 16b reveals finer, elongated, and deeper dimples, indicating that the addition of Sc enhances the plasticity of the composite, resulting in higher ductility. However, as the GNP content increases, the number of dimples on the fracture surface decreases, suggesting that the strength of the composite becomes primarily reliant on the GNPs. The fracture mechanism shifts to the pullout and rupture of the GNPs. However, at a GNP content of 1.0 wt%, the GNPs tended to agglomerate, exacerbating the separation of the GNPs from the matrix. This reduces the effectiveness of load transfer and ultimately leads to a decrease in the plasticity of the composite.

**Table 2:** Mechanical properties of composites.

Sample	Yield strength (MPa)	Ultimate tensile strength (MPa)	Elongation (%)
0GNPs-U	62.45	235.51	0.90
0GNPs-B	124.6	282.82	2.0
0.2GNPs-T	99.93	326.81	3.20
0.5GNPs-T	92.13	286.44	0.82
1.0GNPs-T	82.66	269.57	0.98

**Fig. 17:**
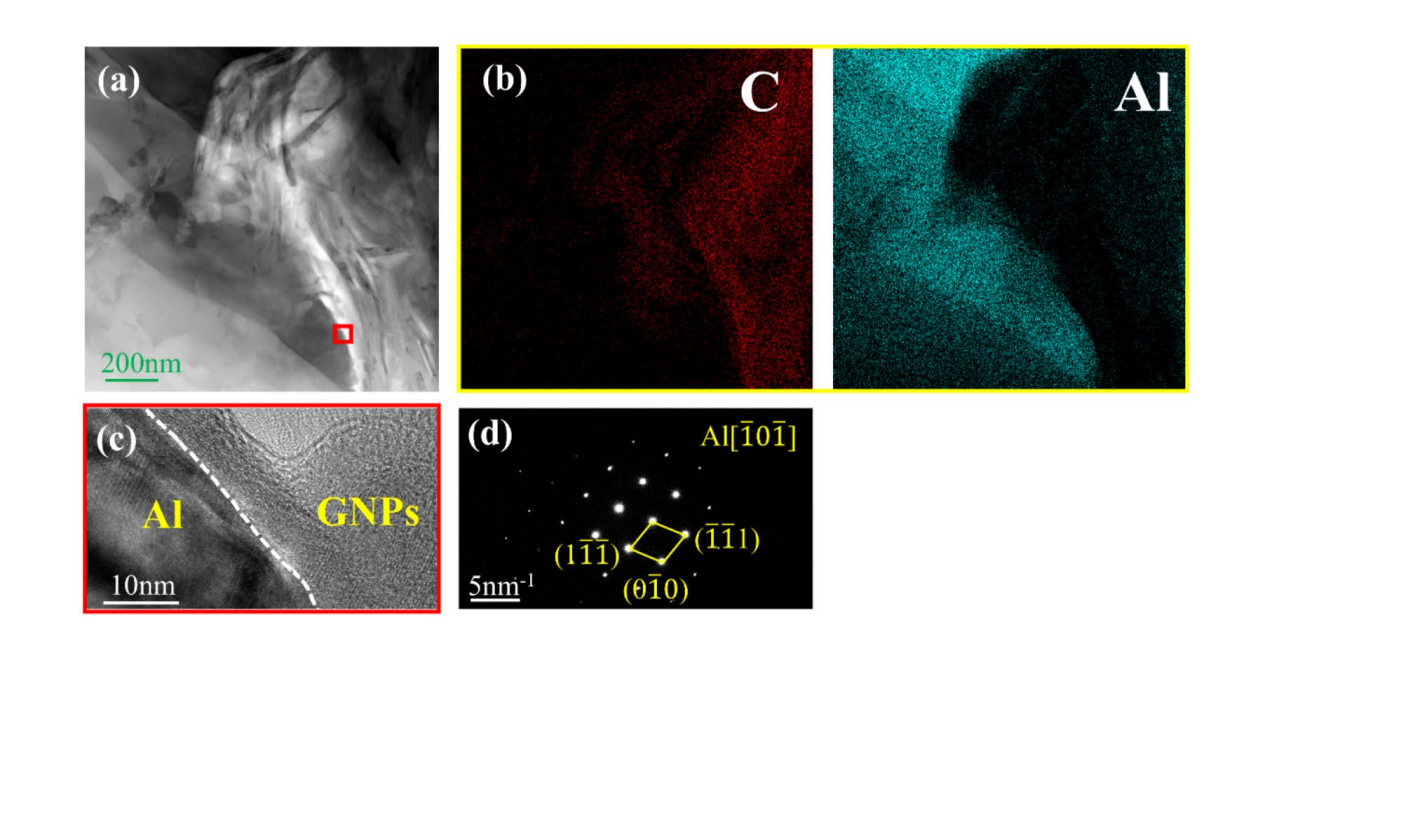
TEM images of 0.2GNPs-T composite: (**a**) TEM bright field image of 0.2GNPs-T composite, (**b**) EDX plot corresponding to (**a**), (**c**) HRTEM image of the red region in (**a**) showing the interfacial structure of the GNPs with the aluminum matrix, (**d**) SAED of Al.

The TEM images of the 0.2 GNPs-T sample are shown in Fig. 17. In Fig. 17a, the distinct morphology of the GNPs is clearly visible. Figure 17b presents the EDX map of Al and GNPs. Figure 17c shows the Al/GNPs interface observed under high-resolution transmission electron microscopy (HRTEM). The HRTEM results reveal a close bond between the GNPs and the Al matrix, forming a non-coherent interface. Figure 17d displays the selected area electron diffraction (SAED) pattern of Al.

**Fig. 18:**
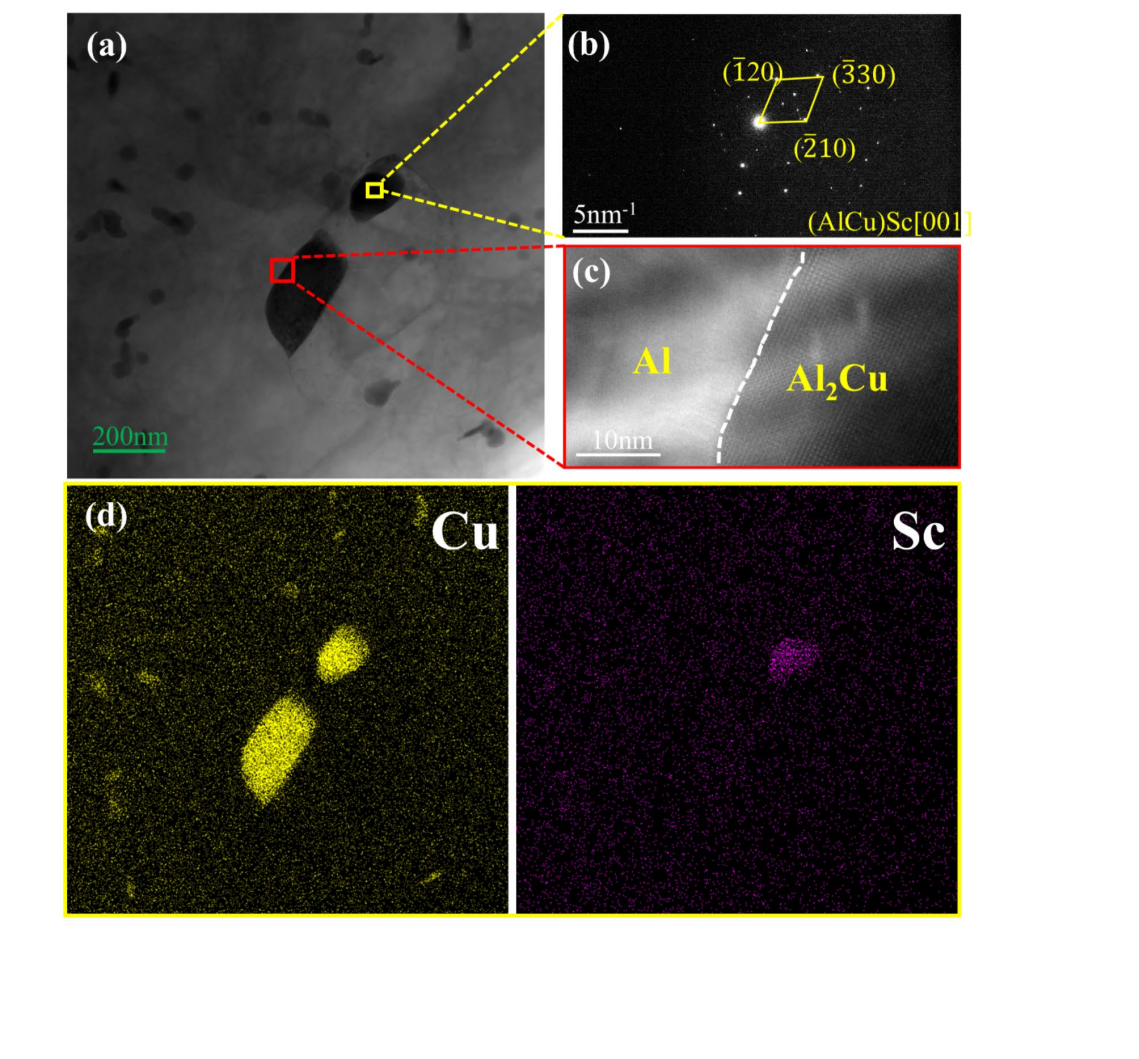
TEM images of 0.2GNPs-T composite: (**a**) TEM bright field image of 0.2GNPs-T composite, (**b**) SAED of (AlCu)Sc, (**c**) HRTEM image of the Al-Al_2_Cu interface, (**d**) corresponding EDX plots of (**a**).

Figure 18 shows the TEM images of the Sc-rich region. As seen in Fig. 18a, d, the Sc element is concentrated in the θ′ phase, which has a size of less than 200 nm, significantly smaller than the 1.31 µm observed in the EBSD results. This demonstrates that Sc plays a role in promoting the refinement of the θ′ phase. Furthermore, SEAD analysis of the Sc-enriched region revealed the presence of AlCuSc particles, as shown in Fig. 18b. Figure 18c presents the HRTEM image of the Al matrix and the Al_2_Cu interface, where the Al matrix and Al_2_Cu interface are well bonded without exhibiting a coherent interface. Figure 18d shows the EDX maps of elements C and Sc for the region in Fig. 18a.

According to the experimental results on mechanical properties, the addition of Sc and GNPs both significantly enhance the yield strength, ultimate tensile strength, and elongation of the material. The primary strengthening mechanisms contributing to the composite’s increased strength include grain refinement, dislocation strengthening, load transfer strengthening, and Orowan strengthening.

According to the EBSD results, the addition of GNPs leads to grain growth in the composite, rendering grain refinement ineffective. Therefore, this mechanism will not be further analyzed. Based on the theory of shear lag, the primary strengthening mechanism of GNPs in the composite is load transfer. As shown in Fig. 17c, the interface between GNPs and the Al matrix exhibits high bond strength, enabling effective load transfer by the GNPs. The EBSD analysis of the average dislocation density indicates that the addition of Sc and GNPs increases the material’s dislocation density, although the increase is minimal and has little impact on the mechanical properties^39^. Orowan strengthening arises from the interaction between particles (including precipitates) and dislocation motion. Al_2_Cu, the primary precipitate phase in 2024 aluminum alloy, is further promoted by the addition of Sc, which facilitates the precipitation of the intragranular θ′ phase. This reduces stress concentration caused by the θ′ phase, thus promoting the precipitation of the intergranular θ phase. The formation of θ phase impedes dislocation movement, which, in turn, enhances the overall strength of the material.

### Electrical resistivity

In GNPs-Sc/Al2024 composites, the effects of GNPs, Sc, and Al2024 on electrical conductivity vary significantly. Notably, the high conductivity and large specific surface area of GNPs facilitate the formation of a conductive network within the composite, thereby enhancing the overall conductivity. The addition of Sc to aluminum alloys markedly improves their strength, hardness, heat resistance, corrosion resistance, and weldability, although it does not have a direct impact on the conductivity of the composite. To investigate the influence of various factors on the electrical conductivity of GNPs-Sc/Al2024 composites, the effective medium theory (EMT) was applied to assess the resistivity of the composites based on the volume fraction of GNPs, which is described as the following euation^40^.

1 $$\:\begin{array}{c}{\rho\:}_{composite}={\rho\:}_{max}\times\:\left[\frac{1+\frac{2{\rho\:}_{max}}{{\rho\:}_{GNPs}}-2f\times\:\left(\frac{{\rho\:}_{max}}{{\rho\:}_{GNPs}}-1\right)}{1+\frac{2{\rho\:}_{max}}{{\rho\:}_{GNPs}}+2f\times\:(\frac{{\rho\:}_{max}}{{\rho\:}_{GNPs}}-1)}\right]\end{array}$$

Where $\:{\rho\:}_{composite}$, $\:{\rho\:}_{max}$ and $\:{\rho\:}_{GNPs}$ are the electrical resistivity for the composite, Al2024 matrix and GNPs, respectively, $\:f$ is the volume fraction of the GNPs particles in the composites.

Dislocations, grain boundaries and nanoparticles significantly influence the electrical resistivity of aluminum-based composites. The impact of microstructural factors on the electrical conductivity (EC) of the composites can be assessed according to the Matthiessen’s law, which expresses the resistivity of the composites as follows^41–43^:

2 $$\:\begin{array}{c}{\rho\:}_{composite}={\varDelta\:\rho\:}_{max}+\varDelta\:{\rho\:}_{dis}+\varDelta\:{\rho\:}_{gb}+{\varDelta\:\rho\:}_{\rho\:}\end{array}$$

Where $\:{\varDelta\:\rho\:}_{\rho\:},\:\varDelta\:{\rho\:}_{dis}$ and $\:\varDelta\:{\rho\:}_{gb}$ are the increased electrical resistivity induced by GNPs particle-self, generated dis locations due to GNPs particles, generated grain boundaries due to GNPs.

3 $${\Delta \rho _{{gb}} = \frac{2}{3}\rho _{{gb}} \Delta \frac{s}{v}}$$

Where $\:{\rho\:}_{gb\:}$ the specific grain boundary electrical resistivity of Al, S/V is the grain boundary area per volume which could be 2.37/D, if assuming that the grains are tetrakaidekahedral, and D is the grain size.

4 $$\Delta \rho _{{dis}} = 2\sqrt 3 \rho _{{dis}} \Delta \left| {\frac{{FWHM\cdot\cos \theta - \frac{{0.9\lambda _{{cu}} }}{D}}}{{\sin \theta }}} \right|by~XRD$$

where $\:{\rho\:}_{dis}$ is the specific dislocation electrical resistivity of Al, FWHM stands for the full width at half maximum at a certain peak $\:\theta\:$ under an X-ray wavelength of λ (Cu).

The variation in resistivity induced by particles is highly correlated with the particle volume fraction, as expressed in Eq. 5. In the composites studied, the volume fraction of the reinforcing particles ranges from 1 to 2.2%, while the increase in resistivity, $\:{\varDelta\:\rho}_{\rho}$ is only between 1.5% and 3.3% of $\:{\rho\:}_{m}$. Therefore, it can be concluded that the nanoparticles themselves are not the primary factors affecting resistivity.

5 $$\Delta \rho _{\rho } = \rho _{m} \frac{{3f}}{{2\left( {1 - f} \right)}}$$

The Williamson-Hall^44^ equation can be utilized to separate the effects of lattice strain and grain size, thereby allowing for the determination of the lattice strain and grain size of the material, as shown in Eq. 7.

6 $$\:{\rho\:}_{m}={\rho\:}_{max}+\varDelta\:{\rho\:}_{dis}+{\varDelta\:\rho\:}_{gb}$$

7 $$\:{\beta\:}_{hkl}cos\theta\:=\frac{K\lambda\:}{D}+4\epsilon\:sin\theta$$

Where $\:{\beta}_{hkl}$ is the width of the diffraction peak at half its maximum height,$\:\epsilon\:$ stands for the lattice strain.

8 $$\:\epsilon\:=(\frac{\beta\:}{4\text{tan}\theta\:})$$

The relationship between the electrical resistivity of the GNPs-Sc/Al2024 composite and both grain boundary density and grain size are outlined based on the calculations. The resistivity of the composite exhibits a positive correlation with dislocation density and a negative correlation with grain size. This linear relationship suggests that the primary reason for the increase in electrical conductivity is the reduction in dislocation density and the enlargement of grain size. The increase in grain size and the decrease in dislocation density reduce electron scattering. Additionally, the incorporation of GNPs provides more pathways for electron transport, thereby enhancing the material’s conductivity. Table 3 presents precise data on the grain size and high-angle grain boundary content obtained through EBSD analysis. Grain refinement leads to an increase in grain boundary density, which enhances scattering effects and reduces electrical conductivity. As the number of high-angle grain boundaries increases, the electron transport pathways become more complex, further contributing to the decline in conductivity.

**Table 3:** Microstructural data and resistivity of the sample.

Sample	HABs (%)	Grain size (µm)	Electrical conductivity/(%IACS)	Electrical resistivity/×10^− 8^Ω
0GNPs-U	48.37	1.41 ± 0.23	31.08	5.6912
0GNPs-B	44.71	1.31 ± 0.05	39.83	4.3642
0.2GNPs-T	38.95	1.60 ± 0.05	46.95	3.7262
0.5GNPs-T	36.67	2.28 ± 0.18	44.73	3.9716
1.0GNPs-T	59.42	4.97 ± 0.09	43.92	4.0294

Despite the remarkable mechanical and electrical properties of GNPs, high concentrations of GNPs are prone to aggregation, which compromises the homogeneity of the composites and limits its overall mechanical properties and electrical conductivity^44^. When GNPs aggregate, larger clusters form, disrupting the continuity of the material and significantly reducing the mechanical properties in localized regions. This also impedes the conduction paths for electrons, thereby decreasing the electrical conductivity of the composite. Moreover, the aggregated graphene nanoplatelets can introduce stress concentration points within the material, further diminishing its tensile strength and fatigue resistance^45^.

The content of GNPs presents a potential trade-off between strength and electrical conductivity. Understanding this balance is crucial for simultaneously improving both the strength and conductivity of materials, which holds significant implications for the development of high-strength, high-conductivity materials. Therefore, controlling the GNPs content to simultaneously enhance both the strength and conductivity of the material, while maintaining the balance of composite properties, will be a primary focus of our future work.

Although the incorporation of GNPs and scandium Sc into aluminum alloys enhances the material’s mechanical properties, the aggregation of GNPs at higher concentrations can compromise both the strength and the uniformity of the electrical properties. This aggregation of graphene significantly impacts the overall performance of the composite. To address this challenge, we propose employing strategies such as surface functionalization^46^, the use of^47^, and ultrasound-assisted dispersion techniques^48^ to improve the dispersion of GNPs. These approaches aim to enhance the overall performance and stability of the composites.

## Conclusions

In conclusion, different GNPs concentrations of 0.1 wt% Sc/Al-Cu-Mg composites were fabricated using powder metallurgy and vacuum hot-pressing sintering. The GNPs-Sc/Al2024 composite was successfully produced through ball milling, vacuum hot pressing sintering, and multiple passes of hot rolling at 440°C. The incorporation of 0.1 wt% Sc and GNPs enhanced the mechanical properties and conductivity of the Al-Cu-Mg composite. When the mass fraction of GNPs reached 0.2%, the overall performance of the 0.1 wt% Sc/Al-Cu-Mg composite was optimized, yielding a maximum tensile strength of 326.81 MPa, a fracture elongation of 3.2%, a hardness of 112.96 HV, and a conductivity of 46.95 IACS, representing increases of 38.76%, 255.55%, 51.07%, and 51.21%, respectively, compared to the matrix material. The addition of Sc promoted the precipitation of the θ′ phase in the matrix material, thereby enhancing the strength of the Al-Cu-Mg composite. The combined addition of GNPs and Sc can enhance the mechanical properties and electrical conductivity of the material. However, the agglomeration caused by a high content of GNPs may weaken the overall enhancement effect on the material.

## Methods

### Raw materials

Al2024 powder (purity 99.9%, average diameter 10 μm, Beijing Hongyu New Materials Co., Ltd.) with a nominal composition of Al-4.3Cu-1.5Mg-0.5Mn (wt%) was selected as the matrix alloy. Graphene nanoplatelets (GNPs) (3–10 layers, average size approximately 20–30 μm, Nanjing Xianfeng Nano Materials Co., Ltd.) and Sc powder (purity 99.99%, average diameter 75 μm, Enyi Metal Materials Co., Ltd.) were utilized as reinforcing phases. The experimental samples comprised nominal compositions of Al2024, 0.1wt% Sc-Al2024 composite, 0.1wt% Sc/0.2wt% GNPs-Al2024 composite, 0.1wt% Sc/0.5wt% GNPs-Al2024 composite, and 0.1wt% Sc/1.0wt% GNPs-Al2024 composite.

### Fabrication of the composites

Initially, the 2024 aluminum alloy powder and Sc powder were mixed in a container in predetermined proportions and subjected to ball milling for 36 h. Subsequently, GNPs were added, and the milling continued for an additional 12 h at a rotation speed of 31 rpm in an argon atmosphere. Upon completion of the mixing process, the blended powder was packed into a stainless steel mold and sintered using a ZR-6-8Y type vacuum molybdenum wire hot press furnace. The sintering was conducted at a pressure of 50 MPa and a temperature of 610 °C, with a holding time of 60 min and a heating rate of 6 °C/min. The resulting experimental green body had a diameter of 50 mm and a thickness of 28 mm. The green body was then cut into blocks measuring 40 mm × 40 mm × 3 mm and subjected to annealing in a muffle furnace at a temperature of 440 °C for 1 h. This was followed by multiple passes of unidirectional hot rolling with a deformation of 20%, resulting in dense samples with a final thickness of approximately 2.4 mm.

Analysis of composition, microstructure and mechanical properties. The microstructural features, morphology of the composite powder, and tensile fracture surfaces of the experimental samples were examined using a scanning electron microscope (SEM, S-3400 N). The elemental distribution of the samples was determined using an energy dispersive spectrometer (EDS, Xplore30 Aztec One). The EBSD samples were ion polished in the sectioning mode, with a scanning step size set to 0.2 μm. The phase composition of the samples was analyzed with an X-ray diffractometer (XRD, Ultima IV, Japan) under Cu *K*α radiation (0.15418 nm) at a scanning speed of 10°/min, with 2θ scans conducted over a range of 20° to 90°. The microstructure of the composites was characterized using a field emission transmission electron microscope (TEM, JEM F200). In the scanning transmission electron microscopy (STEM) mode, element mapping was performed using energy-dispersive X-ray spectroscopy (EDX).

Microhardness measurements were performed using a DHV-1000 microhardness tester with a load of 100 gf and a loading time of 15 s. For the tensile tests, the experimental samples were machined into dog-bone shapes with dimensions of 42 mm × 16 mm × 2.4 mm using an electrical discharge machining method, ensuring the tensile axis was parallel to the rolling direction. Room temperature tensile performance tests were conducted using a universal testing machine (Zwick/Roell Z020 system) at a strain rate of 1 × 10^−3^ s^−1^. The friction properties of the samples were evaluated using a controlled-atmosphere friction and wear testing machine (WTE-2E), with a GCr15 steel ball as the counter material, a load of 10 N, a reciprocating frequency of 5 Hz, and a friction duration of 30 min. The resistivity of the rolled experimental samples was measured using an eddy current conductivity meter (FET-3 × 3, Fort Electronics, Suzhou, China).

## Data Availability

All data generated or analysed during this study are included in this published article.
